# Electrophysiological correlates associated with contributions of perceptual and conceptual fluency to familiarity

**DOI:** 10.3389/fnhum.2015.00321

**Published:** 2015-06-05

**Authors:** Wei Wang, Bingbing Li, Chuanji Gao, Xin Xiao, Chunyan Guo

**Affiliations:** Beijing Key Laboratory of Learning and Cognition, Department of Psychology, College of Education, Capital Normal UniversityBeijing, China

**Keywords:** familiarity, perceptual fluency, conceptual fluency, FN400, ERP

## Abstract

The present research manipulated the fluency of unstudied items using masked repetition priming procedures during an explicit recognition test. Based on fluency-attribution accounts, which posit that familiarity can be driven by multiple forms of fluency, the relationship between masked priming-induced fluency and familiarity was investigated. We classified pictographic characters into High-Meaningfulness (High-M) and Low-Meaningfulness (Low-M) categories on the basis of subjective meaningfulness ratings and identified the distinct electrophysiological correlates of perceptual and conceptual fluency. The two types of fluency differed in associated ERP effects: 150–250 ms effects for perceptual fluency and FN400 effects for conceptual fluency. The ERPs of Low-M MP-same (items that were preceded by matching masked items) false alarms were more positive than correct rejections during 150–250 ms, whereas the ERPs of High-M MP-same false alarms were more positive than correct rejections during 300–500 ms. The topographic patterns of FN400 effects between High-M MP-same false alarms and Low-M MP-same false alarms were not different from those of High-M hits and Low-M hits. These results indicate that both forms of fluency can contribute to familiarity, and the neural correlates of conceptual fluency are not different from those of conceptual priming induced by prior study-phase exposure. We conclude that multiple neural signals potentially contribute to recognition memory, such as numerous forms of fluency differing in terms of their time courses.

## Introduction

Recognition decisions can be subdivided into two expressions: familiarity and recollection. Familiarity refers to the impression that a particular item was encountered previously without the recall of relevant details. For example, a man looks familiar to us, but we cannot recollect where we met him. Recollection, in contrast, implies that the spatiotemporal context or other details about the prior event can be recalled, such as the man’s name or the color of the jacket he was dressed in when we encountered him (for review, see Yonelinas, 2002). Within the literature on event-related potentials (ERPs), a popular view is that familiarity and recollection can be doubly dissociated through specific ERP components known as FN400 (mid-frontal old/new effect) and LPC (late positive complex) respectively (e.g., Curran, 2000; Curran and Cleary, 2003; Rugg and Curran, 2007). However, some researchers suggest that FN400 potentials indicate conceptual priming that co-occurs with familiarity during recognition tests (e.g., Voss and Paller, 2007; Voss et al., 2010).

When meaningful and verbal stimuli such as words and nameable pictures are employed in an experiment (Curran, 2000; Curran and Cleary, 2003), FN400 potentials indicates familiarity; while nonverbal stimuli such as faces and squiggles are utilized (MacKenzie and Donaldson, 2007; Voss and Paller, 2007), FN400 potentials do not correlate with familiarity. Why do FN400 potentials fail to correlate with familiarity in studies using low meaning stimuli? To reconcile with the controversy that FN400 potentials indicate familiarity in some studies or conceptual priming in others, one can resort to the relationship between the familiarity and fluency (Lucas et al., 2012; Lucas and Paller, 2013). Researches linking fluency and recognition memory abound and many reveal that subjects are more likely to judge fluent stimuli as more familiar (or “old”) in a recognition test (Jacoby and Whitehouse, 1989; Whittlesea et al., 1990; Westerman, 2001; Westerman et al., 2002; Whittlesea, 2002; Kurilla and Westerman, 2008; Olds and Westerman, 2012). In their study, Jacoby and Whitehouse (1989) expounded the fluency-attribution account and found that when participants erroneously attributed the enhanced fluency to prior encounter, they tended to report the test item as studied (this could occur for both studied and unstudied items).

Jacoby and Whitehouse (1989) did not clarify whether fluency affected recollection or familarity; however, subsequent studies that manipulated fluency through priming or other means had shown that the enhanced fluency often leaded to recognition judgments based on familiarity (Rajaram, 1993; Miller et al., 2008; Woollams et al., 2008; Andrew Leynes and Zish, 2012; Lucas et al., 2012). The effect of fluency on familiarity was often attributed to perceptual fluency (e.g., Johnston et al., 1991; Rajaram, 1993). However, some studies suggested that conceptual fluency could also contribute to familiarity (e.g., Rajaram and Geraci, 2000; Wolk et al., 2004). Indeed, findings using individual-difference and lesion-mapping approaches (Wang et al., 2010; Wang and Yonelinas, 2012) suggested that the two phenomena shared an underlying mechanism when familiarity was based on conceptual stimulus dimensions.

Virtually, fluency has different varieties and there are many ways to manipulate them (for review, see Alter and Oppenheimer, 2009). For instance, one can employ stimuli in high or low contrast (Reber et al., 1998; Unkelbach, 2006), with different visual or perceptual clarity (Whittlesea et al., 1990; Reber and Schwarz, 1999), or with different typography (Jacoby and Hayman, 1987; Roediger and Blaxton, 1987), and one can use predictive or non-predictive sentence stems (Whittlesea and Williams, 2000, 2001; Wolk et al., 2004). Nevertheless, the most common manipulation is repetition priming first utilized by Jacoby and Whitehouse (1989).

Importantly, it should be noted that priming has various subtypes driven by dissociable forms of fluency. Take repetition priming for visual stimuli as an example. Priming for repeated, physical features of stimuli forms the basis of perceptual priming, whereas priming for meaning of stimuli, independent of physical properties, constitutes the foundation of conceptual priming. Stimuli such as words and nameable pictures can give rise to both types of priming: perceptual priming for the visual word form and conceptual priming for word meaning (Voss et al., 2010). Indeed, Henson (2003) found that the specific regions of the human brain associated with priming-related effects were determined by the type of stimulus and the way it is processed. Therefore, it can be reasonably inferred that the neurocognitive basis of familiarity also depends on the stimulus specifics because familiarity can sometimes derive from fluency that drives priming. In a recent study, Lucas and Paller (2013) suggested that the stimulus specifics on which familiarity was based could influence its neural correlates. In addition, some studies using other paradigms, such as recognition without identification (Ko et al., 2013) and clarity manipulation (Andrew Leynes and Zish, 2012) also provided new insight into the relationship between familiarity and other forms of memory (e.g., priming).

Briefly, knowledge of the relationship between familiarity and fluency can help us approach the above controversy over the neural correlates of familiarity. As various types of fluency, such as conceptual fluency and perceptual fluency have different neural bases, and fluency can contribute to familiarity, there are different neural bases underlying familiarity. In addition, this account can accommodate findings that FN400 effects correlate with conceptual priming. In a recent study, Lucas et al. (2012) proposed a hypothesis that FN400 reflected a conceptual fluency-related precursor to familiarity. That is to say, FN400 effects correlate with familiarity because familiarity is derived from conceptual fluency.

The present research seeks further evidence to clarify these issues by using a unique type of stimuli: ancient Chinese characters, including oracle bone scripts and bronze inscriptions. These stimuli were first used in a memory study conducted in our laboratory (Hou et al., 2013) and the present research adopts the same stimulus set. We seek to identify electrophysiological correlates of familiarity in situations wherein its source can be conceivably tied to fluency induced by masked-priming methods. Our research strategy extends that used by Lucas et al. (2012) which reasoned that the proper way to investigate the relationship between masked priming and familiarity should be to compare ERPs of false alarms with correct rejections. The stimuli used in the present research for memory testing were subdivided as a function of meaningfulness, i.e., High-Meaningfulness (here termed High-M) and Low-Meaningfulness (here termed Low-M). The Low-M items induce perceptual fluency and the High-M items induce both perceptual fluency and conceptual fluency. Thus, both conceptual fluency and perceptual fluency can be observed in one experiment. Although there are many experiments about perceptual or conceptual fluency, few studies manipulate these two varieties of fluency within the same experiment (Lanska et al., 2014). A previous study (Voss et al., 2010) using minimalist geometric shapes referred to as “squiggles” showed that neural processing accompanying conceptual priming was distinct from that accompanying perceptual priming (FN400 potentials *vs*. P170 potentials, respectively). We therefore predicted that ERPs related to perceptual fluency would occur earlier than FN400 effects associated with conceptual fluency. We also predicted that when familiarity was driven by different forms of fluency, distinct ERPs will be exhibited.

## Materials and Methods

### Participants

Seventeen subjects (aged 19–26 years, 12 females) participated in the experiment. All were right-handed and reported normal or were corrected to normal vision. Data from one subject was collected but excluded due to excessive ocular artifacts and/or electrode drift (>25% of trials). All subjects signed an informed consent and were paid for their participation. None of the subjects majored in History or Ancient Chinese Language and none had any background in learning Chinese oracle and bronze inscriptions. This research was approved by the Human Research Ethics Committee at Capital Normal University.

### Materials

**Figure 1:**
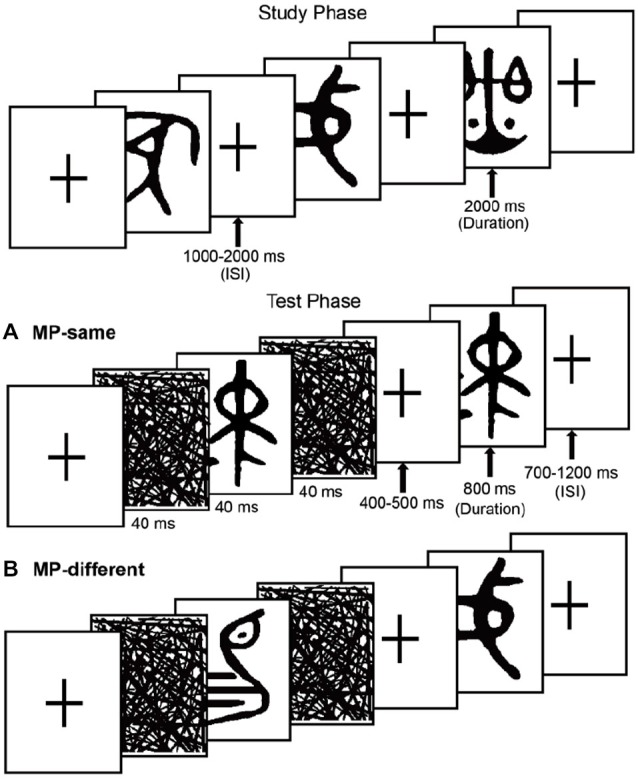
**Schematic representation of the experiment, showing examples of the stimuli**. The task in the study phase was to rate meaningfulness. The test phase task was to make recognition judgments. **(A)** MP-same trials, the unstudied test items preceded by matching masked primes. **(B)** MP-different trials, the test items preceded by nonmatching masked primes.

The stimuli consisted of 600 ancient Chinese characters. An additional 25 items were used as fillers. These hieroglyphs cannot be understood by contemporary people without professional knowledge of history and the ancient Chinese language (see Figure 1). All items were presented in black on a white background at central fixation and subtended approximate visual angles of 4.1°vertically and 3.4°horizontally. The MP-same/MP-different status of the stimulus sets were counterbalanced across participants.

### Procedure

The experiment included 5 study-test blocks. In each study phase, 40 items were presented in a random order bounded by filler items (two primacy buffers and two recency buffers). In each test phase, participants completed a recognition test in which 40 studied items were presented again, along with 80 novel items in pseudo-randomized order (no more than three items of the same old/new type occurred consecutively). Participants were misinformed that the ratio of old to new items was 1:1. During each test phase, 50 new trials were MP-same (i.e., a new item that was preceded by a masked presentation of the same item), and 30 new trials were MP-different (i.e., an item that was preceded by masked presentation of a different new item that occurred in the same block). All old trials were MP-different.

Masks took the form of random lines the same size as the test stimuli. One forward mask and one backward mask sandwiched each prime item in the test phase. Participants were not informed about the presence of the masked items. They were only told that these “flickers” would be used to obtain a baseline measure of brain activity. It was emphasized that in order to have the best memory performance, they needed to focus on the test items. Before the formal experiment, there was a practice section for participants to familiarize themselves with the procedure.

Each study trial began with a fixation cross, followed by a 2000 ms study item and then a fixation cross. The ISI was randomized between 1000 ms and 2000 ms. The task was the same as a previous study conducted in our lab, participants were instructed to rate the meaningfulness level of each item using a 4-point scale, with: 1 = highly meaningful, 2 = relatively meaningful, 3 = relatively meaningless, 4 = almost meaningless. Instructions were to press 1 if an item looked like nameable objects, animals or scenes, 2 if it evoked an intangible meaning or connotation, 3 if it only evoked minimal meaning with effort, or 4 if the item could not evoke any meaning. For example, the third hieroglyph in the top row in Figure 1 looked like a “Balance”, so it would be rated as “highly meaningful”. Participants were also told to remember the items for the upcoming memory test.

The test phase followed the study phase in each block after a break of 30 s, during which participants counted backward by threes. Each test phase was preceded by two practice trials (with one new and one old filler item; data not included in analyses). Each test trial began with a fixation cross, the duration of which was randomized between 700 ms and 1200 ms; then a 40 ms forward mask was presented, followed by a 40 ms matching or non-matching prime item, and a 40 ms backward mask. A fixation cross was then shown again, the duration of which was randomized between 400–500 ms, followed by an 800 ms test item and then a fixation cross.

Participants indicated how confident they were in judging an item as studied (or unstudied) by pressing a single button: button 1 = high confidence old (if they were sure that the item was studied); button 2 = low confidence old (if they thought that the item was studied but without confidence); button 3 = low confidence new (if they thought that the item was unstudied but without confidence); button 4 = high confidence new (if they were sure that the item was unstudied). Both speed and accuracy of response were emphasized. Buttons 1–4 corresponded to the index and middle fingers of the right and left hands. The response buttons were counterbalanced between participants.

After the 5 study-test blocks, there was a re-rating phase where participants were asked to rate the meaningfulness of all the items according to the 4-point scale. This allowed meaningfulness ratings to be determined for new items in the experiment. EEGs were not recorded in this phase. Items given meaningfulness ratings of 1 or 2 were categorized as High-M; Items given meaningfulness ratings of 3 or 4 were categorized as Low-M. Thus, the meaningfulness level of old items was rated twice (the study phase and the re-rating phase) while that of the new items was rated once (the re-rating phase). For the sake of consistency, the trials from the test phase were subdivided into High-M and Low-M categories based on the results from the re-rating phase. Given that the meaningfulness ratings for each item were highly variable across subjects, these two categories were created separately for each subject.

After the re-rating phase, participants were asked whether they had noticed something presented during the “flickers”. If the answer was “yes”, they were further asked what they had perceived. In the debriefing, 6 of the 16 participants reported that they had noticed something presented during the “flickers”, and the number of “noticed” trials was less than 10 in a test block. They reported that the “flickers” were ancient Chinese characters similar to the test items. Five of the 6 participants reported that they had not noticed whether the “flickers” and the later test items were the same; 1 of them reported that occasionally the “flickers” ancient Chinese characters were the same as the later test items, and the number of the “same” trials was less than 5 in a test block. Participants were also asked what they imagine the characters as when they rated the meaningfulness levels of the items.

Electroencephalographic recordings were made from 62 scalp sites using Ag/AgCl electrodes embedded in an elastic cap. Electrode locations adhered to the extended international 10–20 system. These electrodes were referenced to the left mastoid during recording and re-referenced to the average of the right and left mastoid offline. Impedance was less than 5 kΩ. EEG signals were filtered with a band-pass of 0.05–40 Hz and sampled at a rate of 500 Hz. Each 1000 ms averaging epoch began 100 ms prior to stimulus onset. Baseline corrections were performed using mean amplitudes of pre-stimulus onset. Trials containing baseline drift exceeding ±75 μV were rejected (*mean* = 3.9%, *SE* = 0.01). EOG blink artifacts were corrected using a linear regression estimate. Repeated-measures ANOVA included Greenhouse-Geisser corrections when necessary. The alpha level was 0.05.

### Analysis Strategy

The behavioral and ERP data were analyzed using repeated measures analyses of variance (ANOVA). The analysis of behavioral results was focused on response time and accuracy. The analysis of ERP results included three steps. First, we investigated ERPs related to fluency from study-phase by comparing the High-M hits with Low-M hits. The mean numbers of artifact-free trials for High-M hits and Low-M hits were 76 and 51. Second, in order to examine the masked priming effects, we collapsed across response types to examine overall differences between MP-same and MP-different unstudied trials for High-M and Low-M items. The mean numbers of artifact-free trials for the four conditions were as follows: High-M MP-same 108, High-M MP-different 78, Low-M MP-same 129, Low-M MP-different 75. Third, we used the unstudied item trials to isolate the neural correlates of familiarity induced by masked repetition priming. The relationship between familiarity and fluency was investigated by comparing false alarms (false recognition) with correct rejections. One participants were removed due to fewer than 16 artifact-free trials for the Low-M MP-different false alarms condition. For the remaining 15 subjects, the mean numbers of artifact-free trials for false alarms and correct rejections were as follows: High-M MP-same 48, 63; High-M MP-different 28, 42; Low-M MP-same 43, 87; Low-M MP-different 25, 51.

Based on the previous research on recognition memory and fluency (Paller et al., 2007; Woollams et al., 2008; Lucas et al., 2012; Hou et al., 2013) and our observation of the results, the ERP amplitudes were averaged for three midline electrode clusters (frontal, F3/Fz/F4; central, C3/Cz/C4; parietal, P3/Pz/P4). Due to low trial counts, all analyses were collapsed across confidence levels.

## Results

### Behavioral Results

#### Response Time

**Figure 2:**
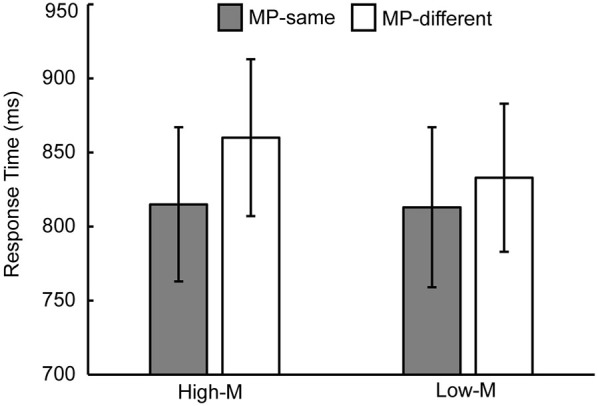
**Response time measures of masked priming effect**. Average response times for MP-same and MP-different stimuli as a function of meaningfulness. The error bars indicate standard error.

Response time in the recognition test for each condition is shown in Table 1. In order to examine the masked priming effect, we collapsed across old/new status and confidence levels of response. For the unstudied items, a 2 (meaningfulness: High-M/Low-M) × 2 (masked priming: MP-same/MP-different) ANOVA was conducted. The main effect of meaningfulness was significant (*F*_(1,15)_ = 8.813, *p* = 0.01). The main effect of masked priming was significant (*F*_(1,15)_ = 21.426, *p* < 0.001), as well as the interaction of meaningfulness and masked priming (*F*_(1,15)_ = 7.97, *p* = 0.013). The simple effect analysis revealed that the masked priming was significant in High-M condition (*F*_(1,15)_ = 37.301, *p* < 0.001) and Low-M condition (*F*_(1,15)_ = 4.91, *p* = 0.043), although the magnitude of masked priming (calculated as RTs of MP-different minus RTs of MP-same for High-M and Low-M respectively) for High-M were greater than that for Low-M (*t*_(15)_ = 2.823, *p* = 0.013). Average response times for MP-same and MP-different stimuli as a function of meaningfulness is shown in Figure 2.

**Table 1:** **Mean RT (ms) in each condition**.

	Studied		Unstudied			
	MP-different		MP-same		MP-different	
	High-M	Low-M	High-M	Low-M	High-M	Low-M
“Old”-high	815 (41)	861 (50)	797 (49)	820 (51)	834 (49)	855 (50)
“Old”-low	866 (58)	859 (53)	851 (58)	860 (63)	911 (75)	880 (61)
“New”-low	835 (60)	836 (61)	839 (61)	825 (60)	881 (59)	865 (60)
“New”-high	841 (52)	807 (45)	797 (49)	791 (47)	855 (45)	806 (44)

*SE in parentheses. △“Old”-high, “Old”-high confidence; “Old”-low, “Old”-low confidence; “New”-low, “New”-low confidence; “New”-high, “New”-high confidence*.

#### Accuracy

Mean percentage of responses in each condition is shown in Table 2. In order to examine the influence of the masked prime on the false alarms, a 2 (meaningfulness: High-M/Low-M) × 2 (masked priming: MP-same/MP-different) × 2 (confidence: high-confidence/low-confidence) ANOVA was conducted. The main effect of meaningfulness was significant (*F*_(1,15)_ = 20.654, *p* < 0.001), reflecting a greater proportion of false alarms for High-M relative to Low-M. The main effect of confidence was also significant (*F*_(1,15)_ = 11.143, *p* = 0.004). The main effect of masked priming was not significant (*F*_(1,15)_ = 0.511, *p* = 0.485). In addition, a marginal significant interaction between meaningfulness and confidence emerged (*F*_(1,15)_ = 3.391, *p* = 0.085). Further analyses indicated that there was a stronger effect of meaningfulness on high-confidence relative to low-confidence false alarms.

**Table 2:** **Mean percentage of responses in each condition**.

	Studied		Unstudied			
	MP-different		MP-same		MP-different	
	High-M	Low-M	High-M	Low-M	High-M	Low-M
“Old”-high	51.3 (4.8)	28.5 (3.8)	17.4 (2.0)	10.7 (1.8)	16.2 (1.9)	10.5 (1.4)
“Old”-low	24.2 (3.3)	24.3 (2.7)	24.7 (2.4)	21.4 (2.2)	23.6 (2.4)	21.8 (1.9)
“New”-low	12.3 (2.5)	21.8 (3.5)	29.7 (3.5)	34.6 (3.7)	30.1 (3.3)	34.3 (4.1)
“New”-high	12.2 (2.1)	25.4 (2.3)	28.3 (2.3)	33.3 (3.4)	30.0 (2.9)	33.4 (3.3)

*SE in parentheses. △“Old”-high, “Old”-high confidence; “Old”-low, “Old”-low confidence; “New”-low, “New”-low confidence; “New”-high, “New”-high confidence*.

### ERP Results

#### Fluency from Study-Phase

**Figure 3:**
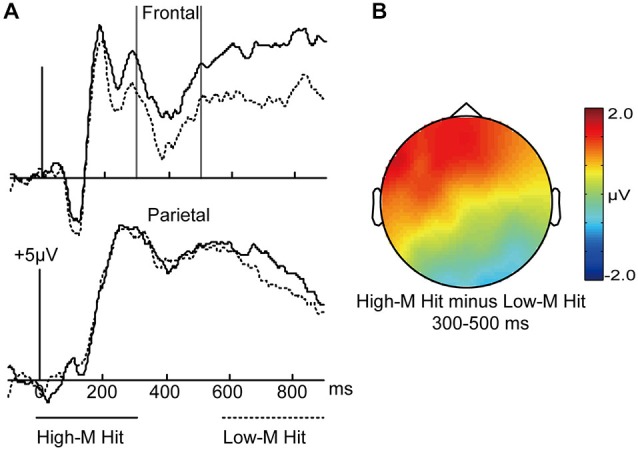
**ERPs for High-M hits and Low-M hits trials. (A)** Waveforms are shown from midline frontal electrodes and parietal electrodes. Gray vertical lines indicate the significant time window (300–500 ms). **(B)** The topographical plot depicts ERP differences between High-M hits and Low-M hits.

In order to investigate the fluency that was induced by study-phase experience, we compared the High-M hits (conceptual fluency present) with Low-M hits (negligible conceptual fluency). *A priori* time window of 300–500 ms was selected according to previous literatures (e.g., Paller et al., 2007; Rugg and Curran, 2007), which is fairly standard for the “FN400”effect. As shown in Figure 3, visual inspection of the grand average waveforms revealed more positive amplitudes from 300 to 500 ms for High-M hits relative to Low-M hits.

A 2 × 3 ANOVA was conducted with meaningfulness (High-M hits/Low-M hits) and cluster (frontal/central/parietal) factors in the 300–500 ms interval, The interaction effect of meaningfulness and cluster was significant (*F*_(2,30)_ = 13.218, *p* = 0.001). The simple effect analysis indicated that the two conditions were significantly different only at frontal (*t*_(15)_ = 2.953, *p* = 0.01).

In sum, we compared the fluency that was induced by study-phase experience and found a typical FN400 effect.

#### ERPs-Perceptual and Conceptual Fluency

**Figure 4:**
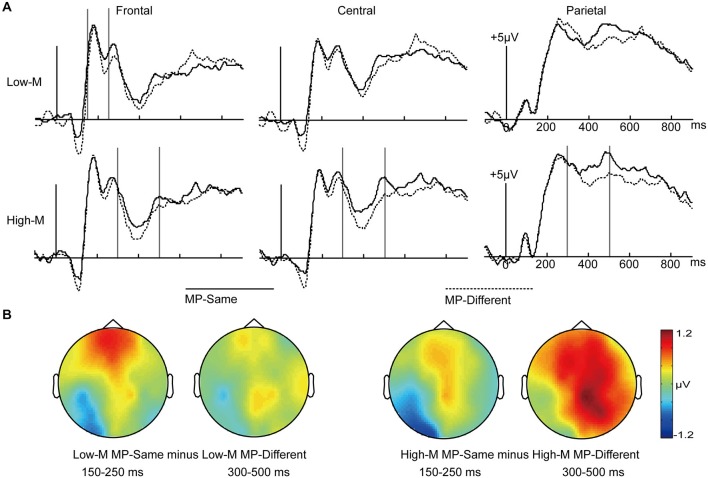
**ERPs for MP-Same and MP-Different trials as a function of High-M and Low-M. (A)** Waveforms are shown from midline frontal electrodes to parietal electrodes. Gray vertical lines indicate the significant time windows (150–250 ms and 300–500 ms). **(B)** The left two topographical plots depict ERP differences between Low-M MP-same and Low-M MP-different; the right two topographical plots depict ERP differences between High-M MP-same and High-M MP-different.

We collapsed across response types to examine the differences between MP-same and MP-different unstudied trials for High-M and Low-M items. As shown in Figure 4, visual inspection of the grand average waveforms revealed that the ERPs differences under these conditions appeared at 150–250 ms interval and 300–500 ms interval. The earlier time window associated with perceptual fluency was selected based on a previous study that used similar masked priming procedure (Woollams et al., 2008).

In order to examine the influence of perceptual fluency, we compared the MP-same with MP-different for Low-M items. We conducted 2 × 3 ANOVA with the factors of masked priming (Low-M MP-same/Low-M MP-different) and cluster (frontal/central/parietal) at intervals of 150–250 ms and 300–500 ms.

For the 150–250 ms interval, the main effect of masked priming was not significant (*F*_(1,15)_ = 1.724, *p* = 0.209). The masked priming × cluster effect was significant (*F*_(2,30)_ = 6.227, *p* = 0.016). The simple effect analysis indicated that the MP-same Low-M trials were more positive than MP-different Low-M trials only in the frontal region (*t*_(15)_ = 2.463, *p* = 0.026).

For the 300–500 ms interval, the main effect of masked priming was not significant (*F*_(1,15)_ = 2.907, *p* = 0.109), and neither was the masked priming × cluster interaction (*F*_(2,30)_ = 0.63, *p* = 0.466).

In order to examine the conceptual fluency induced by masked priming, we compared the MP-same with MP-different for High-M items. We conducted 2 × 3 ANOVA with the factors of masked priming (High-M MP-same/High-M MP-different) and cluster (frontal/central/parietal) at intervals of 150–250 ms and 300–500 ms.

For the 150–250 ms interval, the main effect of masked priming was not significant (*F*_(1,15)_ = 0.23, *p* = 0.638), the interaction of masked priming and cluster was not significant (*F*_(2,30)_ = 0.588, *p* = 0.487).

For the 300–500 ms interval, the main effect of masked priming was significant (*F*_(1,15)_ = 6.653, *p* = 0.021), the interaction of meaningfulness and cluster was not significant (*F*_(2,30)_ = 0.468, *p* = 0.566).

In short, as shown in Figure 4, the influence of perceptual fluency, which appeared at the 150–250 ms interval, was earlier than that of conceptual fluency, which appeared at 300–500 ms interval- consistent with the latency of N400.

#### ERPs-False Recognition

**Figure 5:**
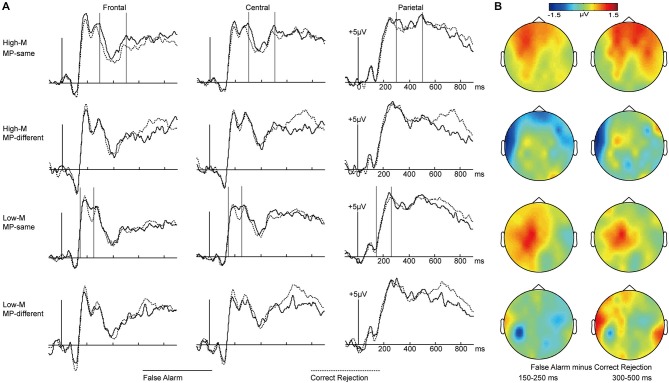
**ERPs for false alarms and correct rejections separately for MP-same and MP-different trials as a function of meaningfulness rating. (A)** Waveforms are shown from midline frontal electrodes to parietal electrodes for four conditions. Gray vertical lines indicate the significant time windows (150–250 ms and 300–500 ms). **(B)** The topographical plots depict ERP differences between false alarms and correct rejections for four conditions at 150–250 ms interval and 300–500 ms interval.

In order to investigate how perceptual fluency and conceptual fluency contributed to familiarity, we compared false alarms with correct rejections separately for MP-same and MP-different at different meaningfulness levels (High-M and Low-M). As shown in Figure 5, visual inspection of the grand average waveforms revealed that the ERPs of false alarms were more positive than those of correct rejections for Low-M MP-same items at the 150–250 ms interval. In contrast, this difference for High-M MP-same items appeared at 300–500 ms.

Formal ERP comparisons across false alarms and correct rejections over the 150–250 and 300–500 ms latency intervals were performed for these four conditions. The 2 × 3 ANOVA were conducted with the factors of response types (FAs/CRs) and cluster (frontal/central/parietal).

For the 150–250 ms interval, the main effect of response types (FAs/CRs) was only significant for Low-M MP-same condition. For the 300–500 ms interval, the main effect of response types (FAs/CRs) was only significant for High-M MP-same condition. The detailed results were summarized in Table 3.

**Table 3:** **The results of repeated measures ANOVA for false recognition**.

	150–250 ms		300–500 ms	
	Main	Interaction	Main	Interaction
High-M MP-S	*F*_(1,14)_ = 0.853	*F*_(2,28)_ = 0.311	*F*_(1,14)_ = 5.996*	*F*_(2,28)_ = 1.559
High-M MP-D	*F*_(1,14)_ = 0.061	*F*_(2,28)_ = 3.067	*F*_(1,14)_ = 0.004	*F*_(2,28)_ = 1.246
Low-M MP-S	*F*_(1,14)_ = 4.905*	*F*_(2,28)_ = 1.181	*F*_(1,14)_ = 2.398	*F*_(2,28)_ = 0.198
Low-M MP-D	*F*_(1,14)_ = 0.173	*F*_(2,28)_ = 0.179	*F*_(1,14)_ = 0.072	*F*_(2,28)_ = 0.329

△High-M MP-S, High-M MP-same; High-M MP-D, High-M MP-different; Low-M MP-S, Low-M MP-same; Low-M MP-D, MP-different. Main, main effect of response types; Interaction, interaction effect of response types and cluster. *p < 0.05.

**Figure 6:**
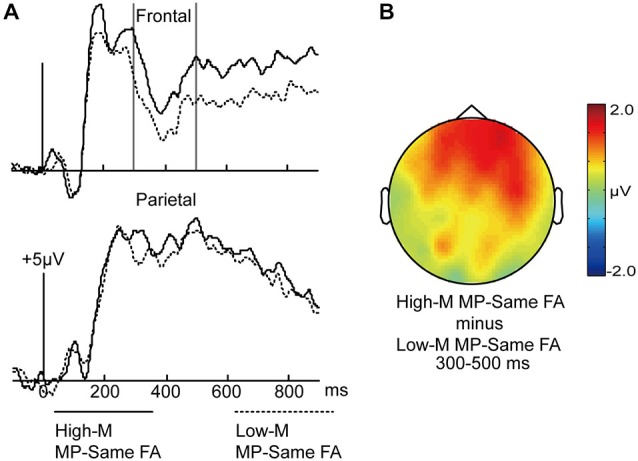
**ERPs for High-M MP-same false alarms and Low-M MP-same false alarms trials. (A)** Waveforms are shown at frontal and parietal electrodes. Gray vertical lines indicate the significant time window (300–500 ms). **(B)** A topographical plot depicts ERP differences between High-M MP-same false alarms and Low-M MP-same false alarms.

In order to examine the differences between perceptual fluency and conceptual fluency as they contributed to familiarity, the ERPs for High-M MP-same false alarms and Low-M MP-same false alarms were compared at 150–250 ms and 300–500 ms intervals (Figure 6). The High-M MP-same items were influenced by conceptual fluency and perceptual fluency, whereas Low-M MP-same items exhibited only the perceptual fluency effect. Therefore, the ERP difference between these categories could be attributed to conceptual fluency.

For the 150–250 ms interval, a 2 × 3 ANOVA with the factors of meaningfulness (High-M MP-same/Low-M MP-same) and cluster (frontal/central/parietal) was conducted. The main effect of meaningfulness was not significant (*F*_(1,14)_ = 0.365, *p* = 0.555), and neither was the interaction between meaningfulness × cluster (*F*_(2,28)_ = 0.075, *p* = 0.822).

For the 300–500 ms interval, a similar 2 × 3 ANOVA was conducted. The main effect of meaningfulness was marginal significant (*F*_(1,14)_ = 4.572, *p* = 0.051). The meaningfulness × cluster interaction was marginal significant (*F*_(2,28)_ = 3.272, *p* = 0.083). The simple effect analysis indicated that the effect of meaningfulness was more robust at frontal.

#### Topographic Analyses

The 300–500 ms positive differences for High-M hits compared to Low-M hits (Figure 3) might index familiarity derived from conceptual fluency. In addition, the 300–500 ms positive differences for High-M MP-same false alarms compared to Low-M MP-same false alarms (Figure 6) might also index familiarity derived from conceptual fluency. We thus performed topographical comparisons on averaged amplitude values from all scalp electrodes after overall amplitude differences were removed (McCarthy and Wood, 1985) to determine if scalp distributions varied across conditions. The distributional differences were indicated by a significant interaction of condition and electrode, although some researchers suggested that such differences did not implicate distinct neural generators (Urbach and Kutas, 2002; Wilding, 2006). The FN400 effects were evident as the ERP difference between: (1) High-M hits and Low-M hits; and (2) High-M false alarms and Low-M false alarms. The analysis aimed to determine if those two FN400 effects had different scalp distributions. However, the electrodes × condition interaction was not significant (*F*_(61,854)_ = 0.623, *p* = 0.679). This result indicated that the scalp distribution of the FN400 effects did not differ between the two conditions.

## Discussion

The present research manipulated the fluency of unstudied items using masked repetition priming procedures during an explicit recognition test. Based on fluency-attribution accounts, which posit that familiarity can be driven by multiple forms of fluency, the relationship between masked priming-induced fluency and familiarity was investigated. We classified pictographic characters into High-M and Low-M categories on the basis of subjective meaningfulness ratings and identified distinct electrophysiological correlates of perceptual and conceptual fluency. The two types of fluency differed in associated ERP effects- 150–250 ms effects for perceptual fluency and FN400 effects for conceptual fluency. The ERPs of Low-M MP-same false alarms were more positive than those of correct rejections during 150–250 ms, while the ERPs of High-M MP-same false alarms were more positive than those of correct rejections during 300–500 ms. The topographic patterns of FN400 effects between High-M MP-same false alarms and Low-M MP-same false alarms were not different from that of High-M hits and Low-M hits. These results indicated that both forms of fluency could contribute to familiarity, and the neural correlates of conceptual fluency were not different from those of conceptual priming induced by prior study-phase exposure.

### Behavioral Results

Response time results indicated that masked priming occurred for the unstudied items; the response time of MP-same items was faster than that of MP-different items, especially for High-M items. The response time of High-M items was faster than that of Low-M items. These suggested that the mechanism behind the masked priming effect was processing fluency.

A difference between our results and those of Lucas et al. (2012) is the absence of masked priming effects on false alarms. The false alarms rate was influenced by meaningfulness and response confidence instead of masked priming. It is difficult to provide an explanation for this discrepancy, particularly given that our procedure was closely aligned with that of Lucas et al. (2012). According to the false alarms rate, we concluded that meaningfulness played a leading role in recognition memory. Possibly, the masked priming effect was covered by meaningfulness.

### Neural Correlates of Fluency Induced by Study-Phase

Some studies suggested that familiarity was indexed by FN400 effect and recollection was indexed by LPC potentials (e.g., Rugg and Curran, 2007). Other studies suggested that familiarity and recollection were both indexed by LPC potentials and the FN400 effect indicated conceptual priming (e.g., Voss and Paller, 2007). The controversy focused on what was indexed by the FN400 effect. The current study obtained typical FN400 effect by comparing the ERPs of High-M (conceptual fluency present) hits with the EPRs of Low-M hits (negligible conceptual fluency). This result suggested that the FN400 effect probably indexed conceptual fluency.

### Neural Correlates of Perceptual and Conceptual Fluency

The influence of perceptual and conceptual fluency was disassociated by time course. Specifically, the ERP differences between MP-same and MP-different Low-M items, which indexed perceptual fluency, appeared from 150 to 250 ms. These findings of perceptual fluency had a similar topography and time window as the study by Woollams et al. (2008). In their study, a main effect of priming was found in an early time window that encompassed the P200 (150–250 ms). This effect was both temporally and spatially dissociable from the FN400 effect, with an earlier onset (150–250 ms) and anterior distribution. Because they used masked repetitions of lexical stimuli, they could not distinguish between perceptual and conceptual contributions to this effect. Yet, the current findings that there were significant differences between MP-same and MP-different for Low-M items during 150–250 ms suggested that this effect indexed perceptual fluency.

These results were different from those of a previous study (Voss et al., 2010) in which perceptual priming was dissociated from conceptual priming via assessing memory for squiggles. In that study, the magnitude of perceptual priming, indicated by faster loop-discrimination responses for old compared to new squiggles, was proportional to the amplitude of frontal P170 potentials. The time window of 150–250 ms effects in the present experiment was different from the P170 potentials. This might be due to the task and stimuli that the present experiment used. For example, the pictographic characters were more complex than squiggles. In addition, perceptual fluency was examined by a direct memory test, rather than an indirect memory test.

The ERP differences between MP-same High-M items and MP-different High-M items, which were primarily caused by conceptual fluency induced by masked priming manipulation, appeared from 300 to 500 ms, consistent with the latency of N400. This effect of conceptual fluency was similar to that of Voss et al. (2010), in which conceptual priming magnitude was proportional across individuals to the amplitude of FN400 potentials, but only for meaningful squiggles. These results suggested that these N400 effects which were functionally identical to FN400 effects should index conceptual fluency.

### Neural Correlates of the Contribution of Fluency to Familiarity

In order to examine the contribution of masked priming-induced fluency to familiarity, we compared the false alarms with correct rejections for the MP-same and MP-different conditions as distinguished by High-M and Low-M. As previously stated, focusing on masked-priming effects on false alarms was advantageous because, on hits trials, processing of retrieved information perhaps interacted with information from masked primes. Hence, relationships between masked priming effects and familiarity experience might be obscured. However, on false alarms trials, retrieval of study-phase information had less influence on brain activity (Lucas et al., 2012).

For Low-M MP-same items, the ERPs differences between false alarms and correct rejections appeared at the 150–250 ms interval, in which only perceptual fluency contributed to familiarity. Thus, this ERP effect probably signaled a contribution of masked priming-induced perceptual fluency to familiarity. For the High-M MP-same items, the ERP differences between false alarms and correct rejections appeared at the 300–500 ms interval (consistent with the latency of FN400), in which both perceptual and conceptual fluency contributed to familiarity. This ERP effect might signal a contribution of masked priming-induced conceptual fluency to familiarity. For MP-different items, perceptual and conceptual fluency did not change. There were no ERP differences between the false alarms and correct rejections at these two intervals. These results suggested that the contribution of perceptual and conceptual fluency to familiarity disassociated in different time courses; namely, familiarity induced by perceptual fluency appeared at 150–250 ms interval, and familiarity induced by conceptual fluency was indexed by the 300–500 ms effect—consistent with the latency of FN400.

We compared MP-same false alarms for High-M (conceptual fluency present) with those for Low-M (negligible conceptual fluency) categories in order to examine ERP effects associated with the contribution of conceptual fluency to familiarity. Similar FN400 potentials to FN400 effects reported above (e.g., High-M hits *vs*. Low-M hits) were obtained, whereas the ERP differences in the 150–250 ms interval were not significant. The scalp distribution of FN400 effects induced by masked priming (High-M MP-same false alarms *vs*. Low-M MP-same false alarms) and those induced by study-phase exposure (High-M hits *vs*. Low-M hits) did not differ from each other. Thus, we found no evidence that the conceptual fluency induced by study phase exposure and that induced by masked-prime have different neural bases.

### Caveat and Conclusion

We did not employ the usual Remember/Know Judgments to dissociate recollection from familiarity and all our analyses were collapsed across confidence levels, leaving open the possibility that participants might have experienced a certain amount of false recollection because of the masked priming manipulation. Although most previous researches suggested that masked priming manipulation primarily affected familiarity but not recollection (e.g., Rajaram, 1993; Rajaram and Geraci, 2000), two studies (Higham and Vokey, 2004; Kurilla and Westerman, 2008) using the parallel ratings remember-know (R/K) procedure found that masked repetition primes affected both recollection and familiarity, and the other two recent studies (Taylor and Henson, 2012; Taylor et al., 2013) using standard R/K procedure found that masked conceptual primes affected recollection, but the effect was only found when conceptual primes occurred in the same experiment with repetition primes. Thus, future studies are expected to determine whether ERPs that are associated specifically with fluency-induced recollection differ from those associated with familiarity.

Finally, by separating materials according to meaningfulness ratings, the present study emphasizes that the experience of familiarity could be driven by both perceptual and conceptual fluency and that the electrophysiological correlates of the contribution of perceptual and conceptual fluency to familiarity are different. Perceptual priming and conceptual priming respectively driven by perceptual and conceptual fluency are processed during different periods. This finding corresponds to theories of multiple memory systems (Tulving, 1972; Tulving and Schacter, 1990; Squire and Zola-Morgan, 1991). However, some researchers maintained that the single-system model could also well account for the dissociations (Berry et al., 2008a,b, 2012). The current findings suggest that multiple neural signals can potentially contribute to recognition memory, such as numerous forms of fluency differing in terms of their time courses and underlying generators (for similar arguments see Lucas and Paller, 2013). The present research indicate that familiarity is not a unitarily determined phenomenon; therefore, in order to acquire a more thorough understanding of familiarity, future studies should take into consideration the multiple contributions of fluency to familiarity. Similarly, when we attempt to explore different memory systems, we should bear their interaction in mind.

## Conflict of Interest Statement

The authors declare that the research was conducted in the absence of any commercial or financial relationships that could be construed as a potential conflict of interest.
